# Electrical resistivity tomography (ERT) and geochemical analysis dataset to delimit subsurface affected areas by livestock pig slurry ponds

**DOI:** 10.1016/j.dib.2022.108684

**Published:** 2022-10-21

**Authors:** Ximena Capa-Camacho, Pedro Martínez-Pagán, Marcos Martínez-Segura, María Gabarrón, Ángel Faz

**Affiliations:** a Department of Mining and Civil Engineering, Universidad Politécnica de Cartagena, 30203 Cartagena, Spain; b Sustainable Use, Management, and Reclamation of Soil and Water Research Group, Escuela Técnica Superior de Ingeniería Agronómica, Universidad Politécnica de Cartagena, Paseo Alfonso XIII, 52, 30203 Cartagena, Spain

**Keywords:** Electrical resistivity tomography, Pig slurry, Geochemical techniques, Pollutants infiltration

## Abstract

The electrical resistivity tomography (ERT) technique was employed with the support of geochemical analyses to delimit the affected surface area by slurry pig ponds. Data were taken in three selected slurry ponds located in Fuente Álamo municipality, Murcia region (SE Spain), to obtain electrical resistivity value-based 2D sections and 3D blocks. All ERT-based survey data were obtained in September 2020 using a SuperSting R8 resistivity meter from Advanced Geosciences Inc. and using the dipole-dipole array consisting of a total of twenty-eight electrodes. The soil samples were taken from drilling core sampling by boreholes at each slurry pond, and physical-chemical analyses of soil samples were obtained using standard laboratory testing methods. Electrical resistivity values and physical-chemical analysis data obtained from soil samples were contrasted, whose comparison showed a correlation between profiles-based electrical resistivity, laboratory-based electrical conductivity (EC) data, and nitrate (N-NO_3-_) content from soil samples. The statistical analysis was run by SPSS Statistics v.23 software (IBM, Neconductivity York, NY, USA) to establish the non-parametric Spearman correlation.

The dataset establishes a reliable methodology and provides insight and information to delimit the affected subsurface area by pig slurry. Data contained within this publication are presented concurrently with Capa-Camacho et al. 2022 [1].

**Specifications Table**

**Table:** 

Subject	Earth and Planetary Sciences, Agricultural Sciences
Specific subject area	Geophysics; 2D/3D electrical resistivity tomography (ERT) technique, Soil science
Type of data	Figure (.jpg). Excel spreadsheet. SuperSting R8 data (.stg) (.dat). Google Earth (.kmz).
How the data were acquired	The SuperSting R8 resistivity meter from Advanced Geosciences Inc. was used for subsurface electrical resistivity data acquisition through Electrical Resistivity Tomography (ERT) surveys outside and inside each pig slurry pond with twenty-eight electrodes and a dipole-dipole array. From each borehole, drilled to a depth of fifteen meters, soil core samples were collected at every remarkable subsurface change. Data from physical-chemical analysis conducted on soil core samples were obtained using standard laboratory testing procedures. Values obtained in the ERT were contrasted with those obtained in the laboratory to determine the correlation at the same deep. The statistics analysis was conducted with the software SPSS Statistics v.23 (IBM, New York, NY, USA) to establish the non-parametric Spearman correlation.
Data format	Measured Raw Analyzed Filtered Interpreted
Description of data collection	The ERT-based electrical resistivity data, providing the subsurface electrical resistivity distribution in Ohm.m, were acquired with the SuperSting R8 resistivity meter from Advanced Geosciences Inc. in three slurry ponds (labeled as No.1, No. 2, and No. 3) in the Fuente Álamo Municipality (Spain) in September 2020. ERT external and internal profiles and electrode interspacing were set up regarding the geometry and conditions of each studied slurry pond. The external ERT profiles were labeled with the “Ep” code, and the internal profiles with the “Ip” code, followed by the order in which they were taken in each slurry pond. Soil samples were collected from each borehole at every meter depth. Each borehole was drilled to a depth of 15m. Soil analyses were conducted to determine moisture content, pH, and electrical conductivity (EC) value. Soluble salts were quantified using a Methrom 850 Professional by IC by ion chromatography. Total nitrogen (TN) content was measured using a CHN 628 elemental analyzer by Leco, and the particle size distribution was obtained using a Mastersizer 2000lf laser diffractometer by Malvern Instruments.
Data source location	• Institution: Universidad Politécnica de Cartagena • City/Town/Region: Fuente Álamo / Murcia • Country: Spain • Latitude and longitude for acquired ERT data: Slurry pond No. 1: 37°39′49.80′′N 1°10′17.99′′O Slurry pond No. 2: 37°45′14.77′′N 1°12′17.08′′O Slurry pond No. 3: 37°40′53.20′′N 1°18′20.42′′O • Latitude and longitude for collected samples: Borehole No. 1: 37°39′50.05′′N 1°10′17.90′′O Borehole No. 2: 37°40′53.24′′N 1°18′0.63′′O Borehole No. 3: 37°45′16.00′′N 1°12′17.38′′O
Data accessibility	Repository name: Mendeley Data Data identification number: (DOI: 10.17632/fr7n8bcybc.5) Direct URL to data: https://data.mendeley.com/datasets/fr7n8bcybc/5
Related research article	Capa-Camacho, X.; Martínez-Pagán, P.; Martínez-Segura, M.A.; Gabarrón, M.; Faz, Á. Delimiting Pig Slurry Affected Subsurface Areas by Combining Geophysical and Geochemical Techniques. Water 2022, 14, 1872. https://doi.org/10.3390/w14121872

## Value of the Data

• The dataset obtained from ERT-based survey, as well as from geochemical analyses, helps to delimit the affected subsurface area by pig slurry infiltration.
• Geophysicists working on similar studies concerning subsurface pollution by intensive livestock activity could benefit from this dataset as a starting point for implementing an appropriate methodology and comparing it with their data obtained in pig slurry infiltration studies.
• The data help decision-makers and final practitioners prioritize the suitable location to be drilled for potential subsurface pollution and near-surface aquifer assessment.
• The data obtained from geophysical and geochemical techniques contribute to establishing a reliable and scalable methodology for studying similar slurry storage structures.

## 1 Data Descriptions

### 1.1 Location of slurry ponds

**Fig. 1:**
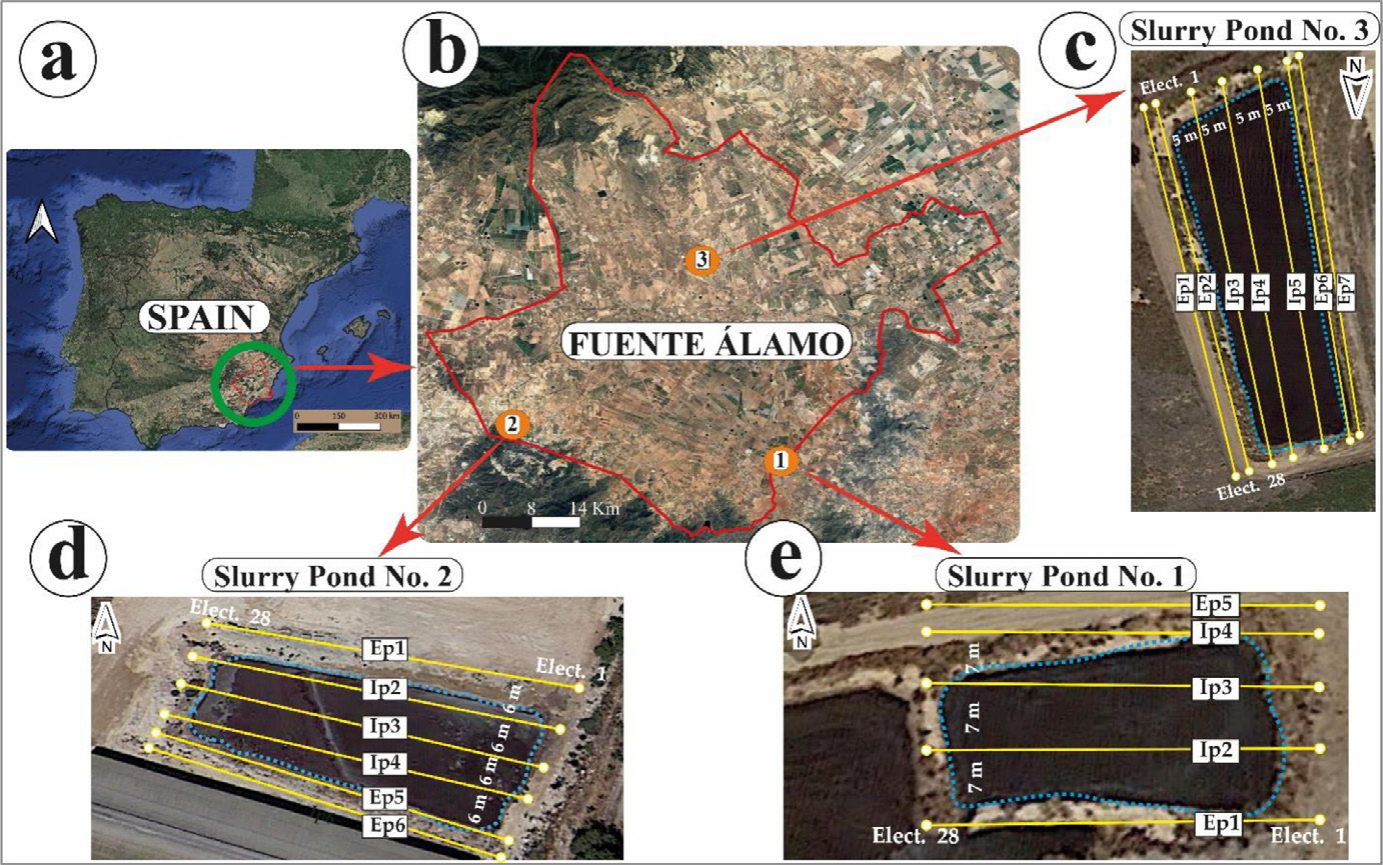
(a) Map of Spain showing the study area. (b) Location of slurry ponds in the municipality of Fuente Álamo. (c) Location of Slurry Pond No 1. (d) Location of Slurry Pond No 2. (e) Location of Slurry Pond No 3. In each slurry pig is indicated the location of internal and external profiles. (Source: Modified from Capa-Camacho et al., 2022) [1].

Data were collected in three slurry ponds in Fuente Álamo municipality, Murcia region (SE Spain). These slurry ponds were chosen in three different soils, thus analyzing the other behaviors of slurry in the different soils. The slurry ponds were located in different lithologies on the geological map of the Region of Murcia [2]. Fig. 1 shows the location of slurry ponds in the municipality of Fuente Álamo, the position, and the separation of parallel external and internal profiles at each studied slurry pond. It is worth noting that the geographic location of each slurry pond, as well as the borehole positioning, are available in the Mendeley repository under .kmz format [3].

**Fig. 2:**
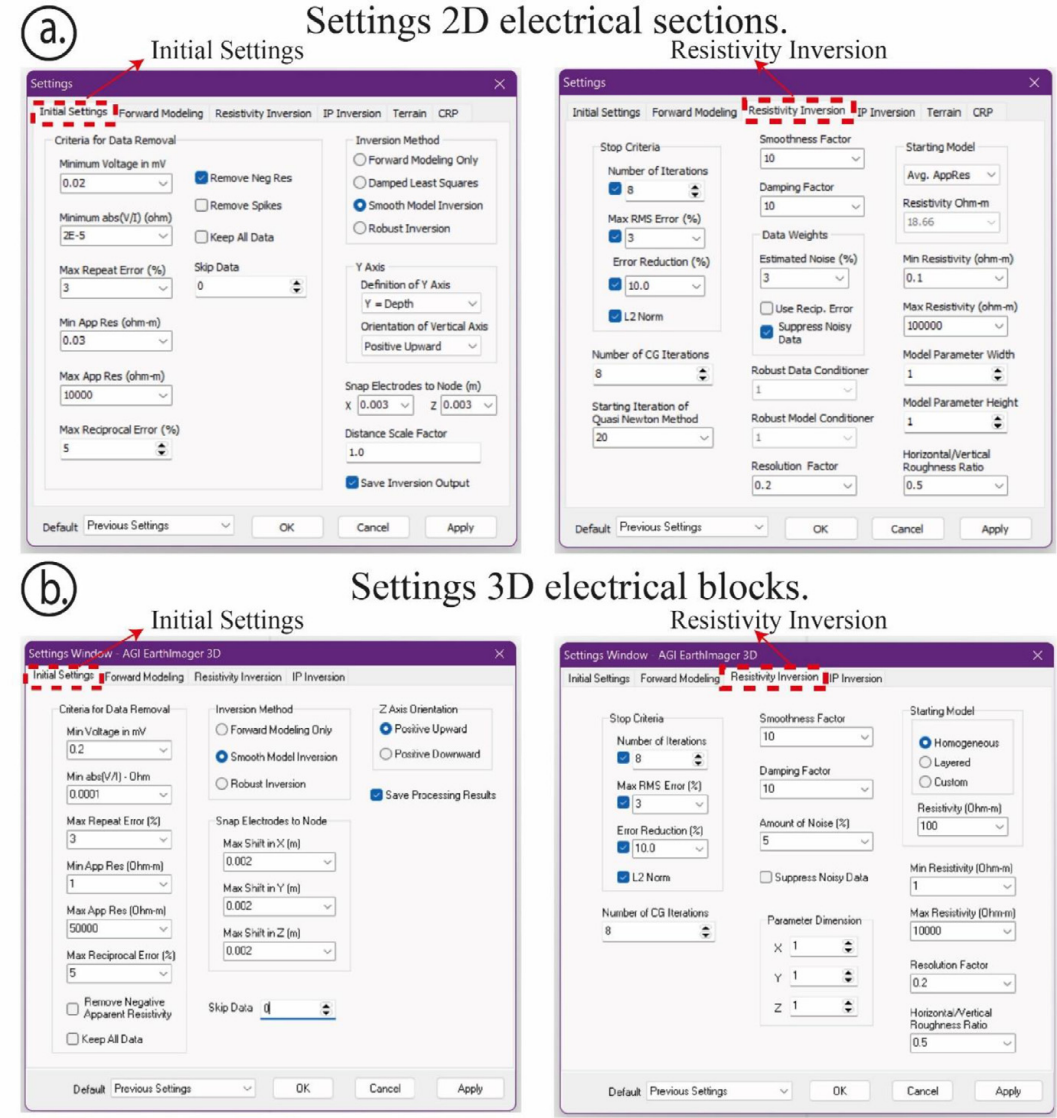
Screenshots of the settings used for the inversion process: (a) Initial and inversion process settings for ERT 2D electrical sections; (b) Initial and inversion process settings for ERT 3D electrical blocks.

**Fig. 3:**
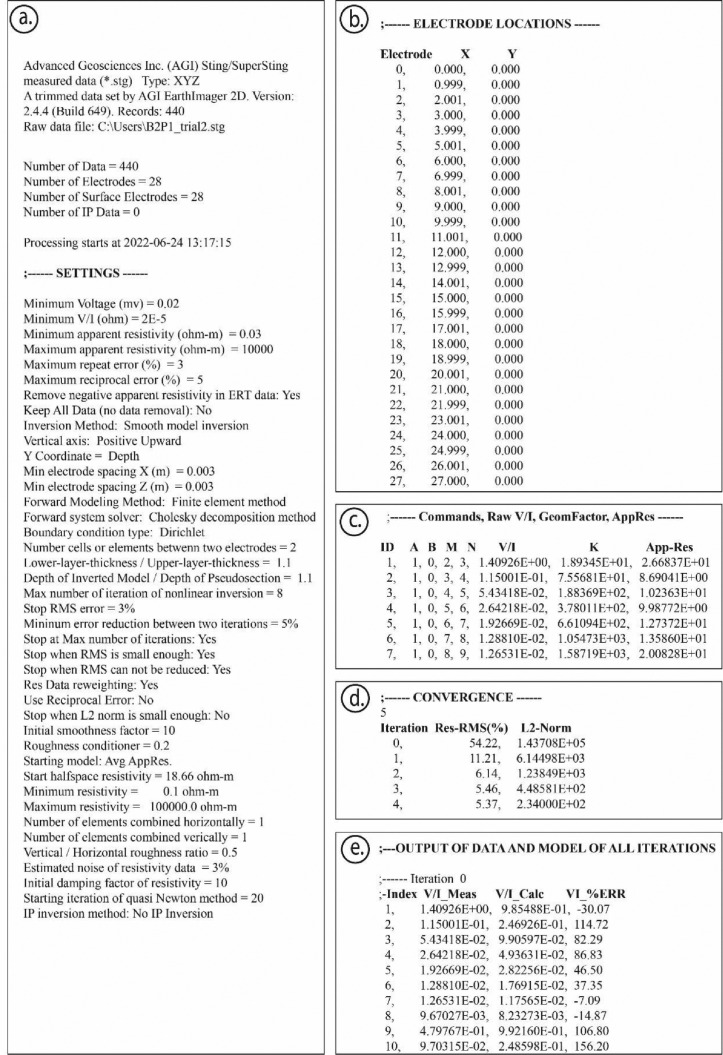
Screenshots of the main data that are provided under .dat and stg format: (a) implemented settings for inversion process; (b) electrode locations; (c) electrodes used for each acquisition (A, B; M, N), employed voltage to current intensity ratio (V/I), geometric factor (K), and apparent resistivity (App-Res); (d) achieved convergence and iteration number associated with their RMS % and L2-Norm values; (e) data and model output of all iterations.

### 1.2 Data of electrical resistivity tomography

ERT survey data were acquired under .stg file format, which provides the following main variables: subsurface distribution of the apparent electrical resistivity (in Ohm.m) value, injected electrical current (in mA), the measured potential difference (in mV), and the chosen electrode separation (in m).

**Fig. 4:**
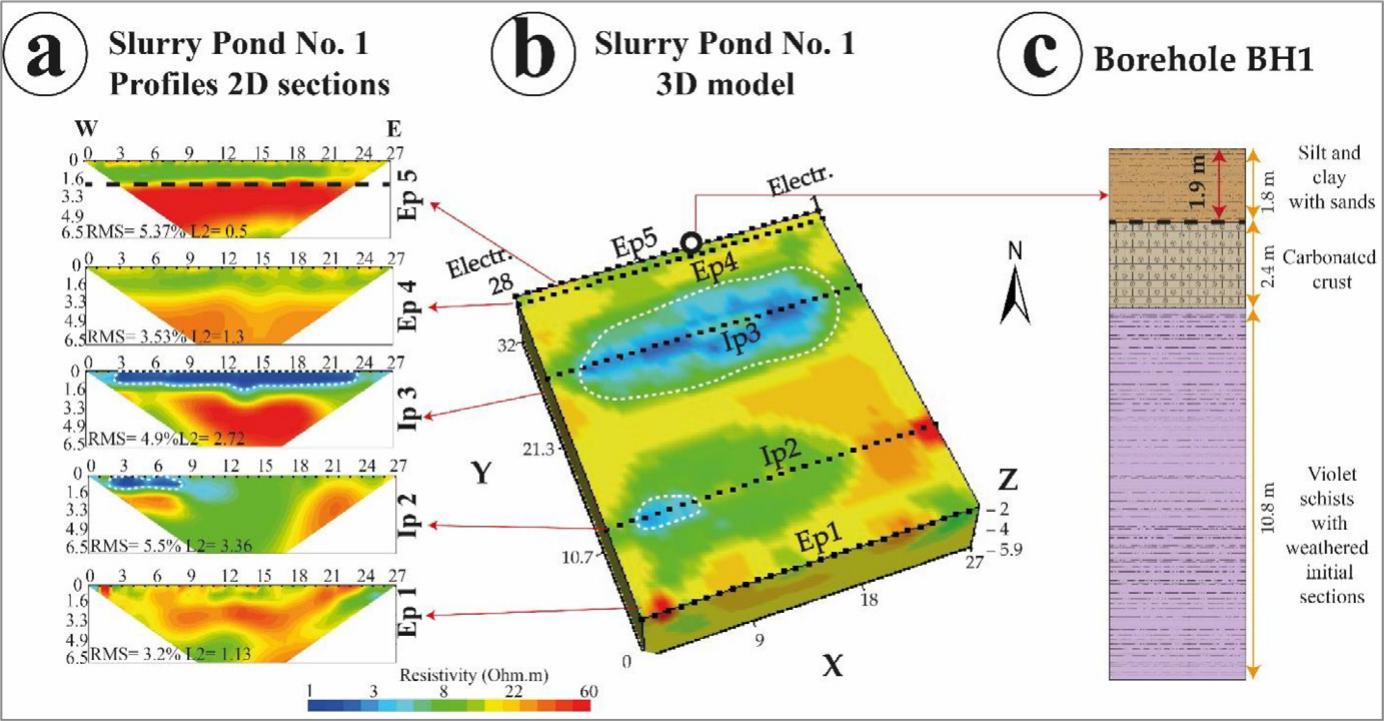
Slurry Pond No. 1 (a) 2D sections obtained from internal and external ERT profiles, (b) ERT 3D block at the slurry pond indicating the borehole position as well, and (c) associated lithological column. (Source: Modified from Capa-Camacho et al., 2022) [1].

**Fig. 5:**
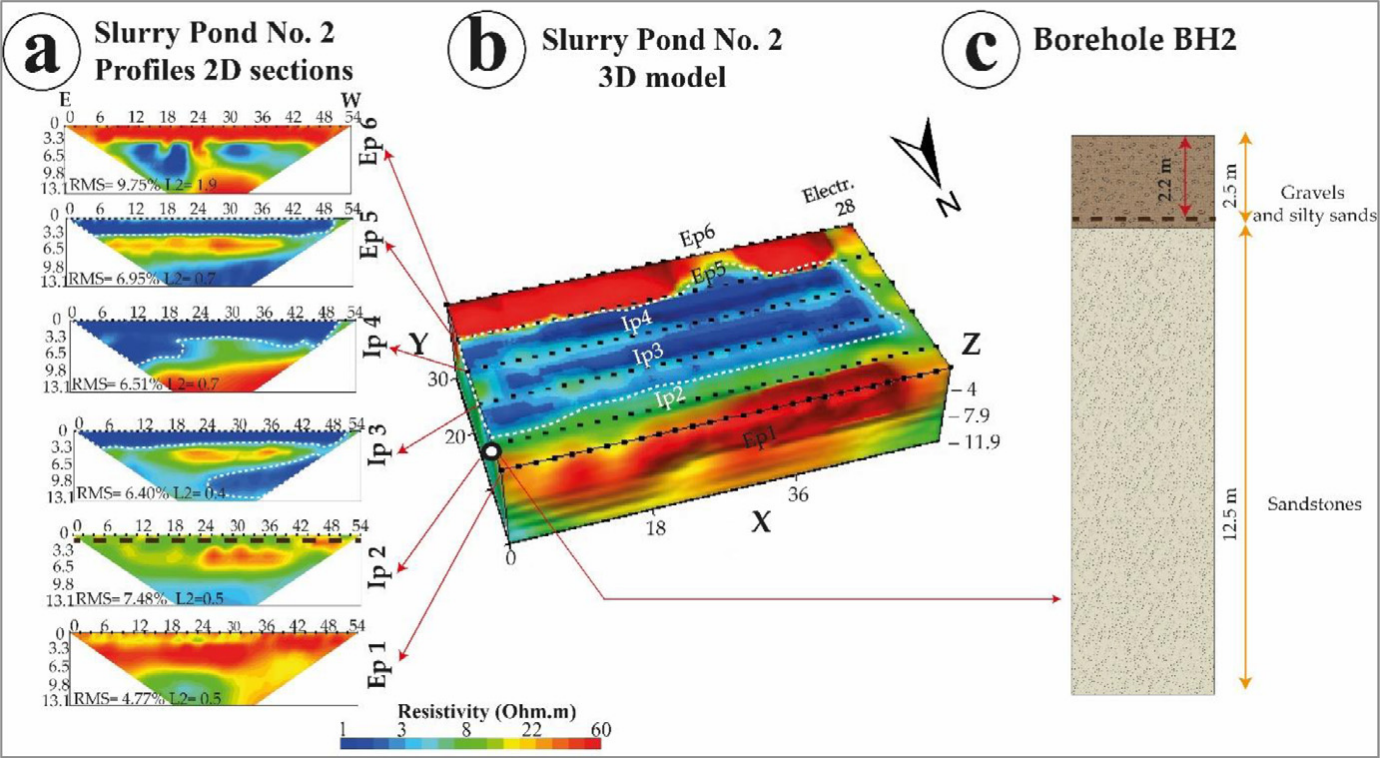
Slurry Pond No. 2 (a) 2D sections obtained from internal and external ERT profiles, (b) ERT 3D block at the slurry pond indicating the borehole position as well, and (c) associated lithological column. (Source: Modified from Capa-Camacho et al., 2022) [1].

**Fig. 6:**
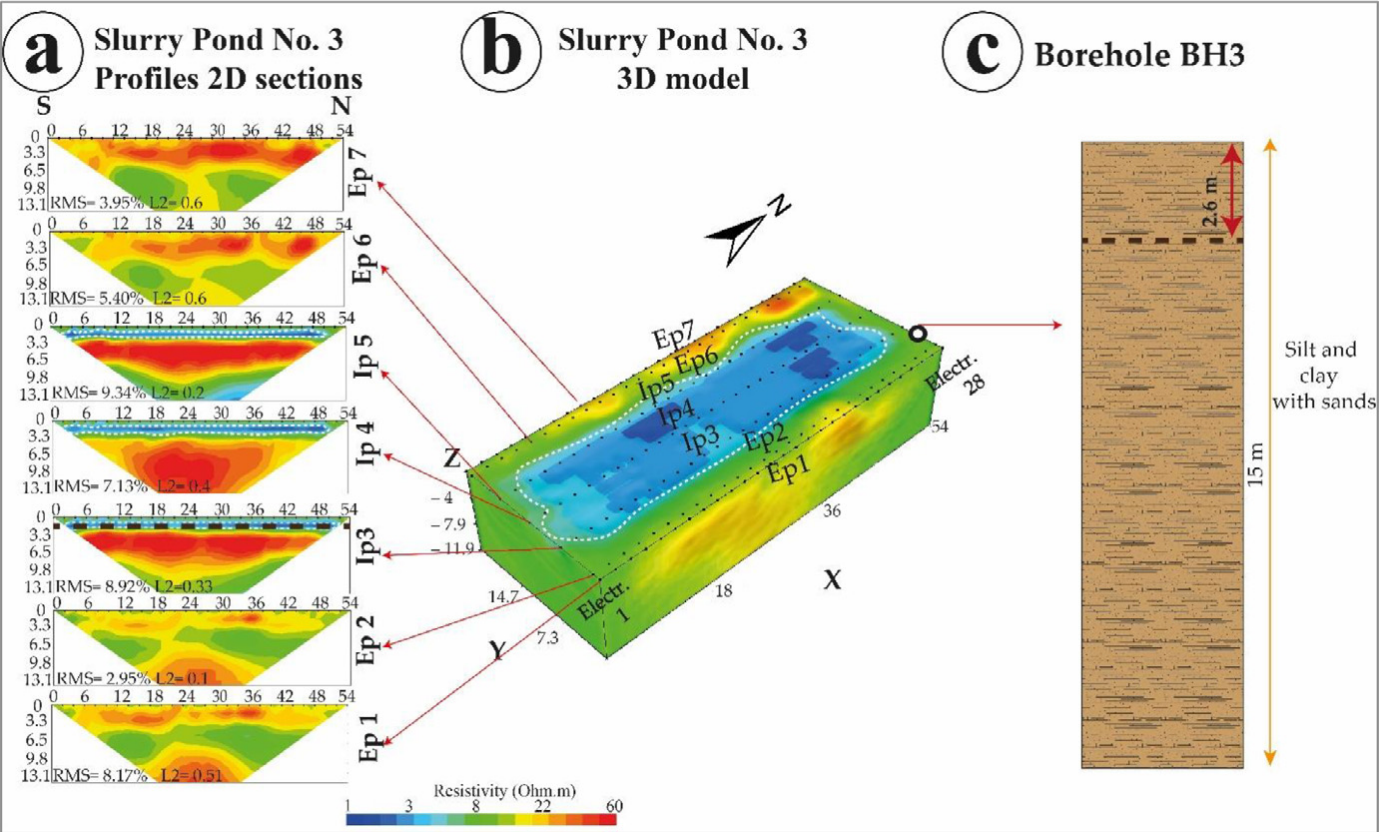
Slurry Pond No. 3 (a) 2D sections obtained from internal and external ERT profiles, (b) ERT 3D block at the slurry pond indicating the borehole position as well, and (c) associated lithological column. (Source: Modified from Capa-Camacho et al., 2022) [1].

These .stg format-based data were processed under EarthImager 2D/3D software package for inversion processing (Fig. 2, Fig. 3), and obtaining the final 2D/3D electrical resistivity models obtained under .out format files. The inversion was based on smooth model inversion with a minimum of eight interactions, and a root means square (RMS) error below 10%. Inversion images show ERT 2D electrical sections and ERT 3D electrical blocks (Figs. 4, 5, and 6).

Fig. 2 depicts screenshots showing the basic settings used for the inversion process through EarthImager 2D/3D software package. In this way, Fig. 2(a) depicts the initial and recommended sets and those settings for resistivity inversion to generate ERT 2D electrical sections through EarthImager 2D software. On the other hand, Fig. 2(b) depicts the initial and suitable settings and those settings for resistivity inversion to generate ERT 3D electrical blocks through EarthImager 3D software.

Those raw and final inverted ERT data are provided under .dat format to be processed through other commercial and/or Python-based open-source geophysical inversion and modeling software, such as pyGIMLi [4] and BERT [5], for the final ERT 2D/3D model comparison and testing. In fact, Fig. 3 shows the main data structure constituted after the inversion process. This structure provides the most relevant information in terms of subsurface electrical resistivity variation to predict underground affected regions by pig slurry. Regarding this information, .dat format files and .stg format files as well, consisting of the description of chosen settings (Fig. 3a), implemented electrode location on the ground (Fig. 3b), electrodes used for each measurement, in terms of current electrodes (A and B) and potential electrodes (M and N), and the acquired apparent electrical resistivity (App-Res) values, and the geometric factor (K) as well (Fig. 3c), and the convergence and the number of interactions undertaken for any absolute RMS error and L2-Norm (Fig. 3d), and, finally, the data and model output associated with each inversion interaction. As it has been mentioned above, two types of ERT data files are provided [3]: (a) raw ERT data, which are the acquired subsurface apparent electrical resistivity values in Ohm.m (e.g., Ep1.dat and/or Ep1.stg); (b) inverted ERT data, which are the final processed subsurface valid electrical resistivity values, in Ohm.m (e.g., Ep1OUT.dat and/or Ep1OUT.stg). It should be highlighted that the adopted labeling convention is as follows: Ep1 for external ERT profile no. 1, Ip1 for internal ERT profile no. 1, Ep2 for external ERT profile no. 2, Ip2 for internal ERT profile no. 2, etc. (refer to Fig. 1).

These reusable ERT data are available in the Mendeley repository [3], in which someone could access an “ERT data” folder consisting of individualized folders for each slurry pond survey containing every raw and inverted ERT data file.

### 1.3 Data of soil samples

Boreholes were drilled on those high conductivity areas previously delimited by the ERT survey at each pig slurry storage pond. Subsurface samples were gathered at every meter depth to a total depth of 15 m.

Fig. 4 shows slurry pond No 1; Fig. 5 shows slurry pond No. 2 and Fig. 6 shows slurry pond No. 3. Each figure shows 2D sections obtained for each internal and external profile, the 3D block of ERT for each slurry pond with the location of the borehole, and the lithological column determined by the borehole.

The core-sampling information from those boreholes, associated with their laboratory analysis data, is also available in the Mendeley repository [3]. The latter consists of a “DATA.xlsx” file, in which there are individualized tabs for each slurry pond survey, labeled as “SlurryPondNo1”, “SlurryPondNo2”, and “SlurryPondNo3”, comprising the following information: Borehole depth (in m), electrical resistivity values from each ERT profile, in Ohm.m, moisture content (in %), pH values, electrical conductivity (EC), in mS/cm, SO_4_^2−^ content, in mg/kg, Na^+^ content, in mg/kg, Mg^2+^ content, in mg/kg, N-NO_3_^−^ content, in mg/kg, clay content, in %, Silt content, in %, and sand content, in %.

## 2 Experimental Design, Materials and Methods

Three slurry ponds were selected in the municipality of Fuente Álamo, Murcia Region (Spain), which were chosen in three different soils associated with different lithological formations [2]. For the acquisition of Electrical resistivity tomography (ERT) data, the Advanced Geosciences Inc. SuperSting R8 resistivity meter was used. Electrical resistivity tomography (ERT) profiles were performed outside and inside each slurry pond. The external profiles were performed to determine the possible lateral infiltration from each slurry pond. On the other hand, the internal profiles were performed to determine the possible vertical infiltration underneath each slurry pond.

An AGI FlexLite passive electrode cable from Advanced Geosciences Inc was used for the external profiles. A marine cable from Advanced Geosciences Inc was used, adding polyethylene floats with plastic clamps to keep the graphite-made electrodes on the slurry surface. The selected array for the ERT survey was dipole-dipole consisting of twenty-eight electrodes. For Slurry Pond No. 1, the electrode separation was one meter, and for Slurry Pond No. 2 and No. 3 were two meters of electrode interspacing.

On slurry pond No.1, two parallel ERT external profiles (Ep1, Ep5) were conducted with a separation between profiles of one meter and two parallel ERT internal profiles (Ip2, Ip3, Ip4) with a separation of seven meters. At the moment of measurements, the slurry pond was dry with only some areas of slurry and in which the presence of manure was more relevant.

On Slurry Pond No. 2, three parallel external profiles (Ep1, Ep5, Ep6) were conducted with a separation between profiles of one meter and three parallel ERT internal profiles (Ip2, Ip3, Ip4) with a separation of six meters. The slurry pond was filled with pig slurry at the moment of measurements. Finally, on Slurry Pond No. 3, four parallel external profiles (Ep1, Ep2, Ep6, Ep7) were conducted with a separation between profiles of one meter and three parallel internal profiles (Ip3, Ip4, Ip5) with a separation of five meters. The slurry pond was filled with pig slurry at the moment of measurements.

**Fig. 7:**
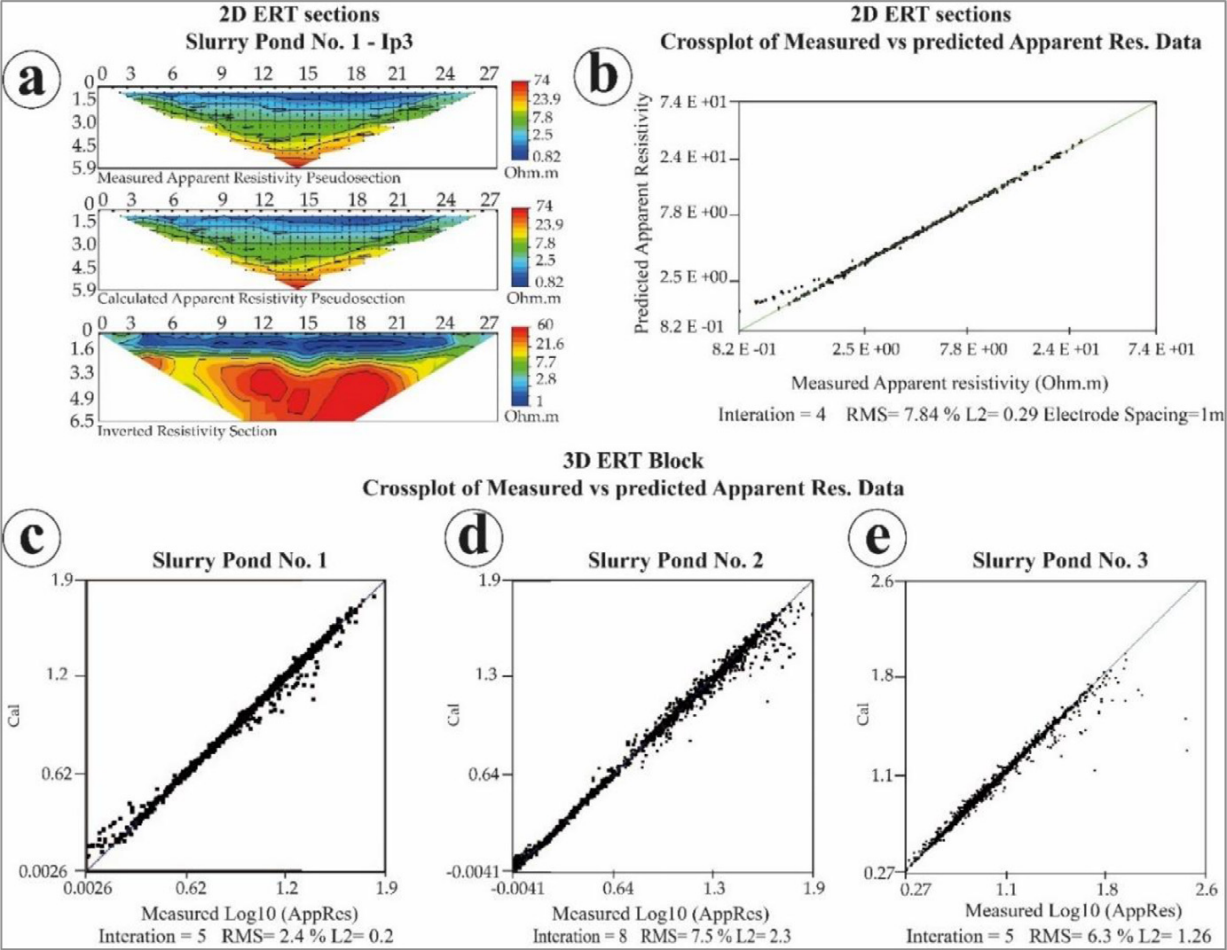
(a) Inversion process to generate the 2D electrical section of ERT profile three at the Slurry Pond No 1. (b) Cross plot of measured vs. predicted apparent resistivity data of the same profile on the left. (c) Cross plot of measured vs. predicted apparent resistivity data of ERT 3D block of the Slurry Pond No 1. (d) Cross plot of measured vs. predicted apparent resistivity data of ERT 3D block of the Slurry Pond No 2. (e) Cross plot of measured vs. predicted apparent resistivity data of ERT 3D block of the Slurry Pond No 3.

Then, 2D electrical sections were obtained from the acquired ERT data, and 3D electrical blocks, as well. This data was processed using EarthImager 2D/3D software package from Advanced Geosciences Inc. This data processing relies on the application of inversion algorithm, which was made with smooth modeling, using a minimum of eight interactions and reaching a root means square (RMS) error of less than 10%. Electrical resistivity values ranged from 1 to 60 Ohm.m. Fig. 7 (a) shows the 2D section profile process of inversion of one profile corresponding to Ip3 of the Slurry Pond No. 1, and 7(b) the cross plot of measured vs. predicted apparent resistivity data with the number of interactions and the RMS obtained for the ERT 2D electrical section. Fig. 7(c), 7(d), and 7(e) depict the cross plot of measured vs. predicted apparent resistivity data with the number of interactions and the RMS obtained for ERT 3D electrical blocks for each slurry pond.

2D electrical sections and 3D electrical blocks as well, pin-pointed an unfavorable zone, in terms of subsurface slurry infiltration, characterized by low electrical resistivity values, which helped to set up appropriately the drilling rig for core-sampling. These core-samples, as it has been indicated above, were submitted to different physical-chemical analyses, as they are described as follows:

The physical-chemical analysis for pH and electrical conductivity values used dried soil samples previously sieved at < 2 mm particle size. pH in a ratio of 1:2.5 w/v (weight/volume) with deionized water measured using selective electrodes [6], electrical conductivity (EC) in a ratio of 1:5 w/v using selective electrodes [7].

Conversely, fresh soil samples were used for moisture and salt content laboratory analysis. In this way, moisture content analysis followed the method proposed by Porta *et al.* [8] soluble salt content analysis. Fresh soil extract was implemented in a proportion of 1:5 w/v. Then the measurements were quantified by ion chromatography using a Methrom 850 Professional by IC.

**Fig. 8:**
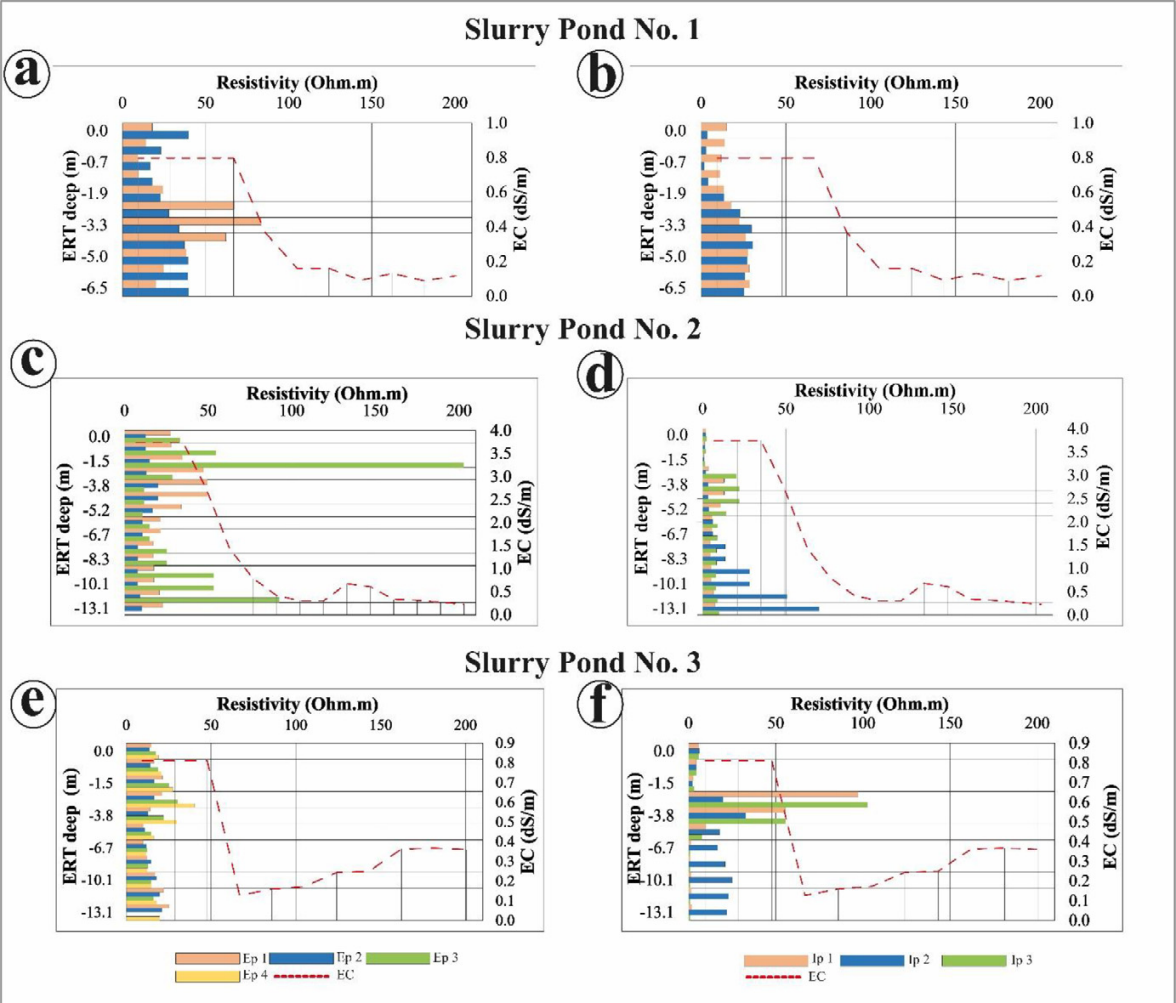
(a), (c), and (e) show the behavior between the ERT external profiles from Slurry Pond No.1, No. 2, and No. 3 and the lab-based EC data at each pig slurry pond. (b), (d) and (f) depict the behavior between the ERT internal profiles from Slurry Pond No.1, No. 2, and No. 3, respectively, and the lab-based EC data at each slurry pond.

**Fig. 9:**
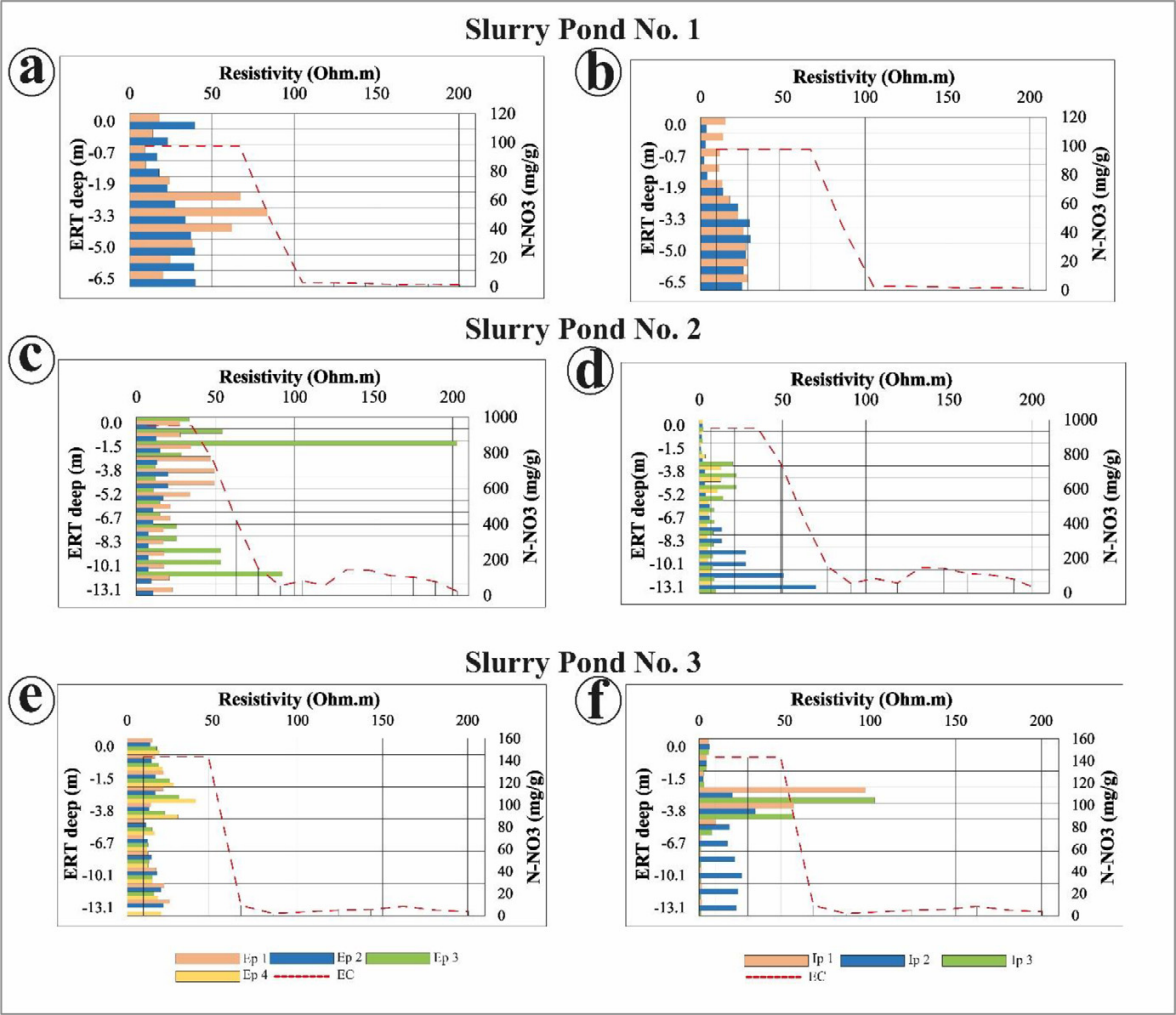
(a), (c), and (e) shows behavior between the ERT external profiles from Slurry Pond No.1, No, 2, and No. 3 and laboratory-based Nitrate (N-NO_3_) content data at each slurry pond. (b), (d) and (f) show the behavior between the ERT internal profiles from Slurry Pond No.1, No. 2, and No. 3, respectively, and laboratory-based Nitrate (N-NO_3_) content data obtained at each slurry pond.

The total nitrogen (TN) content analysis used air-dried ground soil as well, measured using a CHN 628 elemental analyzer by Leco. For the particle size distribution, the soil was pretreated using H_2_O_2_ which allowed the removal of organic matter and the isolation of the mineral particles by means of a Mastersizer 2000lf laser diffractometer by MalvernInstruments. Fig. 8 depicts the behavior between ERT-based electrical data and lab-based subsurface EC data. Similarly, it showed the behavior between the ERT data from internal profiles and the same laboratory-based data of EC. Fig. 9 shows a similar data comparison but uses ERT data from internal and external profiles and laboratory-based nitrate (N-NO_3_) content.

The electrical resistivity values of the internal and external profiles of the ERT at different depths were interpolated and compared with the values obtained from the physical-chemical analysis of soil in the laboratory at the same depth. (Fig. 9) For example, the values obtained by ERT at a 1-meter depth were analyzed with the values obtained at the same depth in the drill cores. Spearman's non-parametric correlation was performed for all data obtained between the ERT values and the values obtained in the soil samples at the same dept using SPSS Statistics v.23 software (IBM, New York, NY, USA).

Spearman's nonparametric correlation allows determining the degree of robustness and the nature of the relationship between the variables of electrical resistivity values of the internal and external profiles of the ERT and the values obtained from the physical-chemical analysis of the soil in the laboratory at the same depth. Table 1 summarizes this comparison generated through the use of SPSS Statistics v.23 software (IBM, New York, NY, USA), which also provides the non-parametric Spearman correlation.

**Table 1:** A correlation of the resistivity values and the physical-chemical analysis of the soil samples. *(Source: Capa-Camacho et al., 2022)*[1].

Profiles	Moisture (%)	pH	EC (dS/m)	Cl^−^ (mg/kg)	SO_4_^2−^ (mg/kg)	Na^+^ (mg/kg)	Mg_2_^+^ (mg/kg)	NO_3_^−^ (mg/kg)	Silt %	Sand %
Slurry Pond No 1										
Ep 1	-	,611*	-,667*	-,668*	-	-,900**	-,797**	-,611*	,639*	-
Ep 2	-	,644*	-	-	-	-	-	-,672*	-	-
Ip 1	-	,919**	-,881**	-,870**	-,835**	-,667*	-,639*	-,919**	,769**	-
Ip 2	-	,802**	-,849**	-,790**	-,737**	-,877**	-,728*	-,793**	,849**	,709*

Slurry Pond No 2										
Ep 1	-	-	-	-	-	-	-	-	-	-
Ep 2	-	-	-	-	-	-	-	-	-	-
Ep 3	-	-	-	-	-	-	-	-	-	-
Ip 1	-,697*	,754**	-,706*	-,780**	-,679*	-,761**	-,725*	-,789**	-	,606*
Ip 2	-,706*	,777**	-,945**	-,890**	-,844**	-,899**	-,734*	-,872**	-	,651*
Ip 3	-	-	-	-	-	-	-	-	-	-
Slurry Pond No 3										
Ep 1	-	-	-	-	-	-	-	-	-	-
Ep 2	-	-	-	-	-	,725*	-	-	-	-
Ep 3	-,779⁎⁎	-	-	-	-	-	-	-	-,730*	,693*
Ep 4	-,642*	-	-	-	-	-	-	-	-,716*	,679*
Ip 1	-	-	-	-	-	-,606*	-	-	-	-
Ip 2	-	,789⁎⁎	-	-	-	-	-,752⁎⁎	-,688*	-	-
Ip 3	-	-	-	-	-	-,775⁎⁎	-	-	-	-

⁎ The correlation is significant at the 0.05 level (bilateral).

⁎⁎ The correlation is significant at the 0.01 level (bilateral).

## Ethics Statements

The authors declare that the present work did not include experiments on human subjects and/or animals, or any data collected from social media platforms.

## CRediT authorship contribution statement

**Ximena Capa-Camacho:** Investigation, Writing – original draft. **Pedro Martínez-Pagán:** Investigation, Visualization, Writing – review & editing. **Marcos Martínez-Segura:** Investigation, Software. **María Gabarrón:** Data curation. **Ángel Faz:** Project administration.

## Declaration of Competing Interest

The authors declare that they have no known competing financial interests or personal relationships that could have appeared to influence the work reported in this paper.

## Data Availability

Dataset1 (Original data) (Mendeley Data).
